# The Human Platelet as an Innate Immune Cell: Interactions Between Activated Platelets and the Complement System

**DOI:** 10.3389/fimmu.2019.01590

**Published:** 2019-07-10

**Authors:** Oskar Eriksson, Camilla Mohlin, Bo Nilsson, Kristina N. Ekdahl

**Affiliations:** ^1^Department of Immunology, Genetics and Pathology, Uppsala University, Uppsala, Sweden; ^2^Linnaeus Center of Biomaterials Chemistry, Linnaeus University, Kalmar, Sweden

**Keywords:** complement, platelets, lectin pathway, disulfides, phosphorylation, thiol isomerases, innate immunity

## Abstract

Platelets play an essential role in maintaining homeostasis in the circulatory system after an injury by forming a platelet thrombus, but they also occupy a central node in the intravascular innate immune system. This concept is supported by their extensive interactions with immune cells and the cascade systems of the blood. In this review we discuss the close relationship between platelets and the complement system and the role of these interactions during thromboinflammation. Platelets are protected from complement-mediated damage by soluble and membrane-expressed complement regulators, but they bind several complement components on their surfaces and trigger complement activation in the fluid phase. Furthermore, localized complement activation may enhance the procoagulant responses of platelets through the generation of procoagulant microparticles by insertion of sublytic amounts of C5b9 into the platelet membrane. We also highlight the role of post-translational protein modifications in regulating the complement system and the critical role of platelets in driving these reactions. In particular, modification of disulfide bonds by thiol isomerases and protein phosphorylation by extracellular kinases have emerged as important mechanisms to fine-tune complement activity in the platelet microenvironment. Lastly, we describe disorders with perturbed complement activation where part of the clinical presentation includes uncontrolled platelet activation that results in thrombocytopenia, and illustrate how complement-targeting drugs are alleviating the prothrombotic phenotype in these patients. Based on these clinical observations, we discuss the role of limited complement activation in enhancing platelet activation and consider how these drugs may provide opportunities for further dissecting the complex interactions between complement and platelets.

## Introduction

### The Complement System

The complement system functions as an intravascular surveillance system and constitutes one of the branches of the innate immune system. It consists of >50 circulating and cell-bound activating proteins and regulators and a number of receptors expressed on vascular cells (1). Most of the plasma-based complement components are synthesized in the liver, although the importance of local production of complement proteins in tissues has recently received renewed interest. The complement system has two major functions; (i) to purge the vascular system of foreign pathogens and substances and (ii) to remove debris and dead cells. It accomplishes these tasks by tagging foreign surfaces with powerful opsonins, by generating potent pro-inflammatory mediators at sites of activation, and by directly lysing targets cells through cell-surface attachment of the membrane attack complex (MAC).

Three major pathways initiate complement activation. The classical and lectin pathways are triggered by pattern-recognition molecules (PRMs) that bind to foreign targets or altered self surfaces, whereas the alternative pathway is continuously activated at a low level in the fluid phase and can be rapidly amplified when an activating foreign surface is present. Each of these pathways converges in the activation of complement component C3, a powerful opsonin and a component of the C5 convertase; this convertase, in turn, activates the terminal complement pathway, leading to MAC assembly by activated C5b, C6, C7, C8, and multiple copies of C9 (C5b-9) (2). Apart from direct lysis of target cells by the MAC, the major mechanisms undergirding the effects of complement are opsonization and generation of pro-inflammatory mediators. C3 and C4 possess a reactive thiol ester group that is buried inside the molecule in the native state but becomes exposed on the surface as a part of a larger conformational change that occurs after activation. The exposed thiol ester allows C4b or C3b to attach to surfaces via a covalent chemical bond, thereby tagging pathogens and debris for clearance by phagocytic cells. A potent inflammatory response is achieved by release of anaphylatoxins, i.e., the N-terminal fragments C3a and C5a that are generated when C3 and C5 are proteolytically activated.

The classical pathway is triggered by C1q, which recognizes the Fc portion of IgM or clusters of IgG fixed to antigens. C1q is also a true PRM in that it directly recognizes foreign surfaces such as patterns of proteoglycans e.g., chondroitin sulfate (serglycin). When C1q binds a target, the C1q-associated proteases C1r and C1s become activated via a conformational change, and they in turn activate C2 and C4, which eventually form the classical pathway C3 convertase. The lectin pathway is evolutionarily older than the classical pathway and has a larger repertoire of PRMs, which likely occurred first as a kind of primitive antibody. These PRMs include three ficolins (ficolin-1, -2, and -3), mannose-binding lectin (MBL) and two collectins (collectin-10 and collectin-11) that recognize pathogen- and damage-associated molecular patterns on foreign microbes and altered self surfaces (3). The lectin pathway PRM genes each encode a monomeric subunit, and these subunits polymerize to form a multimeric protein whose constituent parts are mainly held together via disulfide bonds; this multimerization is necessary to attain sufficient binding avidity for biological activity. Whereas, a single monomer has low affinity for its ligands, the multivalent binding of an assembled multimer leads to high avidity and a binding affinity in the low nanomolar range (4, 5). In an analogous manner to the classical pathway, the lectin pathway PRMs form circulating complexes with serine proteases (MBL-associated serine proteases 1, 2, and 3 [MASP-1, MASP-2, and MASP-3, respectively]) that are activated upon target binding and cleave C2 and C4 to generate a C3 convertase. Ficolin-3 is the quantitatively dominating PRM of the lectin pathway, with a serum concentration of ~39 μg/ml, as compared to <5 μg/ml for ficolin-2, ficolin-1, and MBL and <1μg/ml for the collectins (6) (Table 1). In addition to being the most abundant PRM in the lectin pathway, ficolin-3 is also the strongest complement activator (9).

**Table 1:** Comparison of serum concentrations of MBL and ficolin variants in humans and mice.

**Human gene**	**Gene product (protein)**	**Serum conc (μg/ml) (humans) (6)**	**Mouse gene ortholog**	**Mouse gene product (protein)**	**Serum conc (μg/ml) (mice) (7, 8)**	**Mouse strain**	**-Fold difference: mice vs. humans**
FCN1	Ficolin-1	2.42	FCNB	Ficolin-B	0.130	C57BL/6J	0.05
FCN2	Ficolin-2	2.75^*^	FCNA	Ficolin-A	3.50	C57BL/6J	1.3
FCN3	Ficolin-3	39.9	n/a	n/a		n/a	n/a
MBL2	MBL	1.69	MBL2	MBL-C	45	Serum pool	27
n/a	n/a	n/a	MBL1	MBL-A	7.5	Serum pool	n/a

* *Plasma concentration, human ficolin-2 measurements are unreliable in serum*.

The alternative pathway serves as an amplification loop that can be triggered by C3b generated by the classical or lectin pathways. In addition, as mentioned above there is continuous C3 turnover in the fluid phase as a result of its spontaneous hydrolysis to C3(H_2_O). Although the C3(H_2_O) thiol ester is no longer reactive after hydrolysis, significant conformational changes enable C3(H_2_O) to bind factor B and form a C3 convertase that can initiate the alternative pathway. The structure of C3(H_2_O) resembles that of the proteolytically activated C3b, by analogy to the C3b-like functional properties of C3(H_2_O) (10).

To achieve a selective targeting of foreign surfaces, complement activation is tightly controlled by fluid-phase and membrane-bound regulators, collectively called the regulators of complement activation (RCA) family of proteins. These protect host cells from complement-mediated tissue injury and damage, the hallmarks of diseases caused by insufficient complement regulation. RCA proteins show significant homology and are characterized by repeated domains with a β-sandwich arrangement, so called complement control protein (CCP) or sushi domains (11). Among the membrane-bound regulators, CD46 (membrane co-factor protein, MCP) and CD55 (decay-accelerating factor, DAF) inhibit C3 convertase formation by catalyzing the degradation of C3b by factor I (MCP), or accelerating the decay of any formed C3 convertase (DAF). A third membrane-bound regulator, CD59, acts instead on the terminal complement pathway to limit MAC assembly. These proteins are ubiquitously expressed on most cell types.

Soluble RCA proteins provide an additional mechanism for complement control; they act in the fluid phase or can be recruited to host-cell surfaces. Initiation of the lectin and classical pathways is controlled by the protease-inhibiting C1 inhibitor and C4b-binding protein (C4BP). C1 inhibitor is not specific for complement but is also a physiological inhibitor of the contact and plasma kallikrein systems. Indeed, C1 inhibitor deficiency mainly manifests itself as an overactive bradykinin/kallikrein system and not as a defect in complement regulation (12). The abundant plasma protein factor H serves to regulate the alternative pathway by two mechanisms, either by acting as a co-factor for the serine protease factor I, leading to the proteolytic processing of C3b, or by accelerating disassembly of the alternative pathway C3 convertase.

## Platelets and Complement

### Platelets

Platelets are circulating anucleate cells that form a thrombus and seal a wound when a breach forms in the vascular system. A more integrated view is emerging recently whereby the platelet is seen as a functional immune cell, active in a network with other vascular cells and the cascade systems of blood (13). Platelets are activated by agonists that are located on the injured vessel wall or are generated when coagulation is initiated (14). Von Willebrand factor immobilized on exposed subendothelial collagen binds to GPIb—IX-V on platelets, and trace amounts of thrombin generated during coagulation initiation potently activate platelets through protease-activated receptors (PAR) 1 and 4, G protein-coupled receptors (GPCRs) that are activated through proteolytic cleavage. Thrombin is the most potent platelet agonist known of, and in addition to PARs it also binds the GPIb alpha chain of the GPIb-IX-V complex with high affinity, an interaction which is important for the platelet response to low doses of thrombin (15, 16). Additional platelet agonists that contribute to full activation include adenosine diphosphate (ADP) and thomboxane A2, which both bind to platelet surface GPCRs.

**Figure 1:**
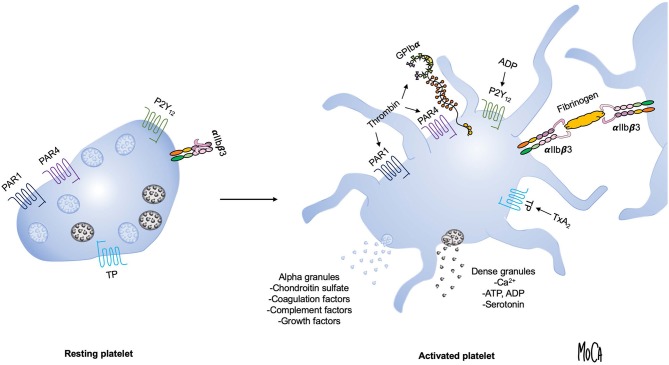
Overview of platelet activation. Platelet activation triggers platelet shape change and granule exocytosis. Alpha granules (blue) contain growth factors, coagulation and complement components and chondroitin sulfate; release of dense granules (gray) provides a source of ADP, ATP, Ca^2+^, and serotonin. Selected platelet agonists and their receptors are indicated. Thrombin binds to GPIbα and activates protease-activated receptor 1 and 4 (PAR1 and PAR4, respectively), ADP activates the P2Y12 receptor, and thromboxane A2 activates the thromboxane (TP) receptor. Agonist stimulation results in integrin α_IIb_β3 activation and the transition to an active conformation that binds fibrinogen and mediates platelet aggregation.

Dramatic changes in platelet morphology and function occur as a consequence of activation in order to rapidly produce a hemostatic plug and, subsequently, a stable thrombus (14). Flip-flop reactions of phospholipids in the platelet membrane lead to the exposure of phosphatidylserine and a negatively charged surface that provide a scaffold for the activation of coagulation factors. The main platelet integrin αIIbβ3 transitions to a high-affinity active state and mediates the formation of a platelet plug by forming cross-links with fibrinogen. A central event in platelet activation is the release of intracellular granules, which serve to amplify platelet activation and create a microenvironment permissive of chemical reactions that are essential for the cross-talk between platelets and protein-based cascade systems. The main types of platelet granules are alpha granules, containing organic macromolecules such as growth factors, coagulation factors, complement components and chondroitin sulfate; and dense granules, which are a source of inorganic compounds, including serotonin, ADP, adenosine triphosphate (ATP), and Ca^2+^. Figure 1 summarizes the key events that occur during platelet activation.

### Platelet-Complement Interactions

Once considered a strictly fluid-phase cascade system, complement's involvement in interactions with vascular cells is now well-appreciated. In the case of platelets, several lines of evidence point to an intimate relationship with complement, with multiple touchpoints that serve to facilitate the host defense response and bridge innate immunity and the intravascular cascade systems. However, if insufficiently controlled, these interactions can result in excessive platelet activation and thromboembolic complications, eventually leading to human disease.

In a number of publications, we have reported our investigation of this cross-talk by using whole-blood experimental systems. As complement activation is dependent on the divalent cations Ca^2+^ and Mg^2+^, any anticoagulant that chelates cations, such as EDTA or citrate, will severely affect complement function. To circumvent this problem, we have developed blood-based experimental systems that use the thrombin inhibitor lepirudin (recombinant hirudin) as anticoagulant, in which the final stage of blood clotting, i.e., the conversion of fibrinogen to insoluble fibrin by thrombin, is prevented (17). Lepirudin has an affinity for human thrombin in the picomolar range and is not known to inhibit any other protease or affect complement function (18). Since thrombin generation is disabled, fibrin generation, and spontaneous platelet activation are prevented, but platelets still can be fully activated by the addition of other agonists.

**Figure 2:**
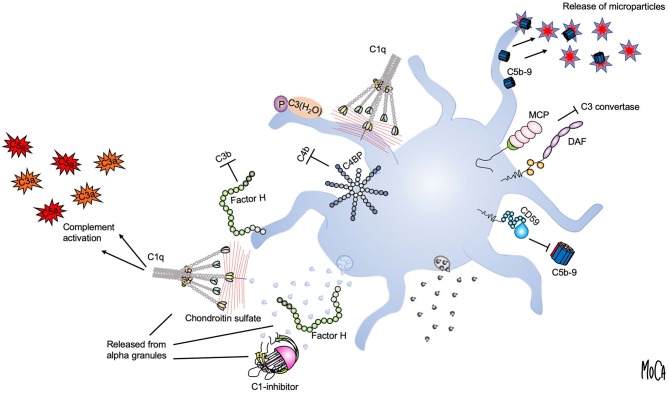
Platelet-complement interactions. Interactions between complement components and activated platelets are shown. The platelet surface provides a binding scaffold for complement factors and supports contact activation of C3 to C3(H_2_O), which interacts with platelet-bound properdin (P) (upper left). C1q recognizes chondroitin sulfate released from activated platelets or bound to the platelet surface and triggers complement activation in the fluid phase (left). Localized formation and surface attachment of sublytic concentrations of C5b9 complexes triggers rapid shedding of pro-coagulant microparticles (upper right). Platelet integrity is maintained by membrane-bound (MCP, DAF, CD59) (right) or fluid-phase complement regulators (C1-inhibitor, factor H, and C4-binding protein (C4BP) (left). C1-inhibitor and factor H are also released from alpha granules (lower left).

Using the lepirudin-based *ex vivo* whole blood system, we found that the glucosaminoglycan chondroitin sulfate, released from platelet alpha granules, acts as a potent trigger of complement activation in the fluid phase (19) (Figure 2, lower right). Activation of platelets leads to the generation of C3a and soluble C5b9 complexes, which can be abolished by adding chondroitinase. We identified C1q as the initiating PRM, given that chondroitin sulfate did not activate complement in C1q-depleted serum, and this failure could be corrected by the addition of purified C1q. Our further studies demonstrated that platelets also recruit a number of complement components to the surface upon activation, and this recruitment is partially dependent on chondroitin sulfate exposure (20–22). In an effort to reconcile these observations with previous studies suggesting that complement activation also occurs on the platelet plasma membrane despite its high expression of complement regulators (23, 24), we reassessed the mechanism by which complement factors bind to platelets. Paradoxically, we found that the platelet surface appeared to be protected from proteolytic complement activation (20). C3 was shown to bind to platelets in the form of C3(H_2_O), but in a manner independent of preceding complement activation. Instead, we posited that the platelet surface provides a scaffold for contact activation of C3 into C3(H_2_O) (Figure 2, upper left). We further showed that platelet-associated C3(H_2_O) can serve as a ligand for CD11b/CD18 expressed on leukocytes to promote the formation of platelet-neutrophil and platelet-monocyte aggregates (25). This finding is in line with results from Saggu et al. (26), who reported that physiological forms of properdin can bind to human platelets after activation and recruit C3(H_2_O) to the surface. Hence, complement components bound to platelets may in some cases serve other functions than that of “classical complement” activation, such as mediating heterotypic interactions with leukocytes.

Although it was not immediately apparent, these findings were quite logical in view of the fact that all vascular cells express high numbers of complement regulators on their surfaces, an essential attribute to allow them to avoid complement-mediated damage. Membrane-integrated complement regulators expressed by platelets include CD46 (27), CD55 (28), and CD59 (29) (Figure 2, right). In addition, platelets bind and recruit soluble complement regulators such as factor H, C4BP, and C1 inhibitor. Both factor H (30, 31) and C1-inhibitor (32, 33) are stored in alpha granules and secreted from platelets, demonstrating a second reservoir of these proteins in addition to the pool circulating in the plasma (Figure 2, lower left). It has been suggested that the released pool of factor H serves to increase the local concentration on the activated platelet surface or in the context of a thrombus. However, the released amount has been quantified and found to constitute only 0.05% of the amount of factor H present in plasma (30), and a substantial amount is bound to the platelet surface (34). Similarly, the released amount of C1 inhibitor is very low when compared to its plasma concentration (33). An interesting possibility is that the platelet-derived pool has distinct functional properties, which would explain why release of such a comparably low amount could be of functional importance.

### Complement and Platelet Microparticles

Another mechanism that may explain the apparent absence of activated complement factors on platelets is the rapid removal of membrane-inserted C5b-9 complexes through the shedding of microparticles (Figure 2, upper right). This process has been proposed as an additional complement-regulatory mechanism by which host cells can maintain membrane integrity when other complement-regulatory mechanisms are inadequate (35). By analogy to this concept, a number of studies from Sims and co-workers have detailed how C5b9 insertion into the platelet membrane can cause platelet activation through intracellular Ca^2+^ elevation and the release of platelet microparticles (36). These microparticles were shown to express binding sites for the FXa/FVa prothrombinase complex and to generate pro-coagulant activity (37). Hence, complement activation that has proceeded to its final stage, i.e., MAC formation, is a strong trigger of platelet procoagulant activity.

Given the apparent absence of complement activation on the platelet surface discussed above, the question remains: To what extent does this mechanism account for platelet microparticle formation under physiological conditions?

C3(H_2_O) bound to activated platelets could potentially combine with factor B to form an alternative pathway C3 convertase to initiate the events leading to MAC formation, a model that has received experimental support (26).

Another potential mechanism for limited complement activation on platelets involves the transmembrane protein P-selectin, a platelet receptor localized to alpha granules that was initially discovered as an activation-specific platelet membrane antigen (38). Its ligand PSGL-1 is expressed on leukocytes, and the P-selectin-PSGL-1 interaction is essential for platelet-leukocyte aggregate formation. Interestingly, P-selectin contains several CCP domains and was proposed to act as a scaffold for complement activation by binding C3b (24). The significance of these observations are not fully determined, and C3 also binds to platelets in a P-selectin independent fashion (20, 26).

An attractive model based on these observations is that the limited C5b9 formed at sublytic concentrations is rapidly shed from the platelet surface in the form of released microparticles, which would maintain platelet integrity and explain the absence of detectable C5b9 at the platelet membrane. Indeed, in other cell types exposed to complement, sublytic C5b-9 concentrations have been shown to induce Ca^2+^ flux and cellular responses such as inflammasome activation concomitant with preservation of membrane integrity (39, 40).

### Evaluation of Cross-Talk Between Complement, Coagulation, and Platelets in Mouse Models

Animal models constitute a valuable tool for studying biological processes in a more physiological context than can be reproduced in a test tube. Recent developments in gene editing technology provide nearly unlimited opportunities to design transgenic mice with specific genetic alterations. However, the use of rodent models to study the role of complement in human disease comes with the inherent limitation that the complement systems of mice and humans, and their immune systems in general, have diverged during evolution as result of adaptation to distinct repertoires of pathogens. There are also important differences in platelet biology between mice and humans, among which the ~3-fold higher platelet count in mice and the absence of the thrombin receptor PAR1 on mouse platelets can be mentioned. In the absence of PAR1, mouse platelets are instead activated by thrombin through a dual PAR3-PAR4 mechanism (41).

Despite these caveats, data from mouse models have lent consistent support to the notion that complement is important for platelet function. Both C3- and C5-deficient mice show impaired venous thrombus formation, albeit as the result of different mechanisms (42). Additional investigations in C3-deficient mice have revealed a modest reduction in PAR4-induced platelet aggregation, impaired thrombus formation *in vivo*, and a markedly prolonged tail-bleeding time (43). Studies have demonstrated the presence on platelets of functional receptors for the anaphylatoxin C3a that mediate platelet activation (44, 45). Since C3a is generated by the cleavage of C3, loss of C3a signaling could contribute to the prolonged bleeding time in C3-deficient mice.

The lectin pathway has recently received attention as a potential amplifying mechanism during thromboinflammation, and we have shown that the lectin pathway is triggered when platelets are activated or when fibrin is formed during blood clotting (22). Intriguingly, in addition to acting on their canonical complement substrates, the MASPs exhibit coagulation factor-like serine protease activity and cleave coagulation factors *in vitro* (46), although it should be noted that these observations have mainly been made using recombinant proteins. In comparison, MASPs exhibit much slower kinetics than true coagulation proteases in cleaving coagulation-related substrates (3). Nonetheless, data from mouse models have indicated that activation of the MBL branch of the lectin pathway can exacerbate atherothrombotic diseases and that pharmacological MBL inhibition is protective against stroke (47), thrombosis (48), and myocardial infarction (49). However, a noteworthy difference between the mouse and human complement systems is the repertoire and abundance of PRMs that initiate the lectin pathway.

Mice have two functional MBL genes, whereas humans have only one. Mouse MBL-C is the ortholog of the human MBL-2 gene, whereas the second mouse MBL variant MBL-A does not exist in higher primates. The circulating concentration of human MBL appears to be around 1.5 μg/ml (6), but the total MBL concentration in mice is >30-fold higher (7). Mice have two ficolins (A and B) that are orthologs of human ficolin-2 and ficolin-1, respectively (50), and their plasma concentrations are similar to those reported in humans (8) (Table 1). It is important to note that ficolin-3, which is the quantitatively and qualitatively dominant lectin pathway PRM in humans (9), does not exist in mice, since the FCN3 gene was inactivated to a pseudogene in the rodent lineage (51). Hence, mice have a strongly MBL-biased lectin pathway, whereas ficolins are the main lectin pathway PRMs in humans.

Population-based studies in humans have provided less conclusive results than have animal models regarding the role of MBL in thrombotic disease. The human MBL2 gene displays extensive genetic variability, and functional MBL deficiency is the most common human immunodeficiency. Genetic analyses have concluded that the high frequency of MBL variants is likely an effect of genetic drift, as opposed to positive selection, and that MBL may therefore be redundant in host defense in humans (52). While the outcome after ischemic stroke is improved in MBL-deficient patients (53), MBL deficiency is associated with an increased risk of arterial thrombosis (54), and myocardial infarction (55). Furthermore, circulating MBL serum concentrations have been found to predict a decreased likelihood of myocardial infarction (56). The favorable outcome associated with the presence of functional MBL in certain epidemiological studies is in apparent contrast to results from mouse models and may suggest an atheroprotective role of MBL in humans, possibly through increasing phagocytic removal of atherogenic vascular debris.

Direct cleavage of C5 by coagulation proteases such as thrombin and factor Xa has been suggested as potential mechanism for activating the terminal complement pathway in the absence of activation triggered at the PRM or C3 level (57). In C3-deficient mice, thrombin has been shown to fully substitute as a C5 convertase, as complement activation occurred normally but was sensitive to thrombin inhibitors. The physiological relevance of this observation for humans has not yet been fully determined, and it appears that mouse C5 is much more susceptible to cleavage by thrombin than that of humans. In general, high concentrations of active coagulation proteases are required for efficient complement factor cleavage *in vitro* (58, 59), and coagulation factors mainly circulate in their zymogen forms *in vivo* at concentrations that are considerably lower than those of most complement components. Furthermore, the ability of endogenous thrombin to cleave C5 has been studied in a human blood-based system anticoagulated with the fibrin polymerization-inhibiting peptide Gly-Asp-Arg-Pro (GDRP), which allows thrombin to be formed but inhibits the formation of a fibrin clot and is fully compatible with complement activation as Ca^2+^ and Mg^2+^ levels are not affected (60). While platelets were rapidly activated and consumed by thrombin in this system, C5a generation was not increased compared to lepirudin-anticoagulated blood, a finding which appeared to refute that thrombin is a physiological C5 cleaving enzyme (60). Similar conclusions were reached from induction of systemic coagulation under sterile conditions in a baboon model, where infusion of factor Xa or phospholipids generated robust thrombin generation as measured by thrombin-antithrombin complex levels, but no measurable increase in complement activation products (61).

## The Role of Allosteric Disulfide Bonds in Platelet Function, Thrombosis and Complement Activation

Chemical modifications of proteins after they have been translated in the ribosome, i.e., post-translational modifications, constitute an important mechanism for regulating their function. Accumulating evidence provides solid support for the concept that such modifications can also occur in the extracellular milieu and that activation of cells provides a microenvironment with sufficient local concentrations of co-factors and a scaffold on which these reactions can be assembled. Here we discuss two such modifications, cleavage of allosteric disulfide bonds and phosphorylation of amino acid side chains, and detail how these two types of modification regulate complement initiation and the role of activated platelets in facilitating these reactions.

The amino acid cysteine has the unique property of being able to form a disulfide bond with another cysteine via its sulfur group. Disulfide bonds stabilize a protein's tertiary and quaternary structure and are formed through an oxidation reaction in proteins destined for secretion during their synthesis and folding in the endoplasmatic reticulum. These reactions are catalyzed by thiol isomerases, a large group of endoplasmatic reticulum-resident enzymes, with protein disulfide isomerase (PDI) being the archetypal member. A general feature of thiol isomerases is their conserved active sites that include a thioredoxin-like fold harboring a Cys-X-X-Cys active site, with two vicinal cysteines separated by two amino acids (denoted X). The two cysteines of the active site can transition between an oxidized and a reduced state and render thiol isomerases capable of catalyzing redox reactions in their substrates (62).

An emerging concept for protein regulation is the modification of so-called “allosteric disulfide bonds,” i.e., a cleavage or formation of a disulfide bond by a thiol isomerase that results in a change in protein function (63). Cleavage of disulfide bonds occurs either through reduction, which requires a source of electrons, or through thiol-disulfide exchange, when the disulfide bond is reshuffled through a reaction with an unpaired cysteine in close proximity (64). In contrast to peptide bonds that are irreversibly cleaved by proteases, disulfide bond cleavage is potentially reversible, since the bond can be reformed through oxidation.

An unresolved question regarding allosteric disulfide bonds is what drives redox reactions in the extracellular milieu. The endoplasmic reticulum contains millimolar concentrations of oxidized and reduced glutathione that constitute a redox buffer that permits efficient disulfide bond formation (65). The cytoplasm is a more reducing environment and features a continuous supply of electrons from NADPH formed during cellular respiration. In contrast, plasma is a comparably oxidizing environment and contains only micromolar amounts of low molecular weight redox pairs such as glutathione and cysteine (66). A possible explanation for these seemingly disparate observations is the ability of platelets to catalyze these reactions, by analogy to the way in which the platelet membrane provides a negatively charged surface for the assembly of coagulation factor complexes during blood clotting. Essex et al. have shown that platelets contain an endogenous glutathione-reducing activity that potentiates platelet aggregation and is hypothesized to originate from intracellular NAPDH (67). In an elegant study, a platelet-targeting redox sensor was devised by combining an anti-CD61 antibody with a quenched reporter (68); this methodology allowed the demonstration of disulfide-reducing activity on the surface on activated platelets in various i*n vitro* systems and during thrombus formation *in vivo*, lending strong support to the concept that platelets provide the necessary conditions for these reactions.

A subset of thiol isomerases can escape the endoplasmic reticulum through a poorly characterized mechanism and are actively secreted by vascular cells. Platelets and endothelial cells secrete a number of thiol isomerases, including PDI, endoplasmic reticulum protein 57 (ERp57), endoplasmic reticulum protein 72 (ERp72), and endoplasmic reticulum protein 5 (ERp5), a group of enzymes that have been termed the vascular thiol isomerases (69). Their presence in platelets and platelet releasates has been confirmed by unbiased proteomic investigations (70, 71). Intense interest in this group was ignited when it was shown that extracellular PDI is essential for thrombus formation and fibrin deposition in various animal models of thrombosis (72, 73). An important role in thrombosis has subsequently been confirmed for other vascular thiol isomerases, including ERp57 (74), ERp5 (75), and ERp72 (76). In addition, the use of chemical inhibitors and blocking antibodies has demonstrated an important role for thiol isomerases in platelet activation, possibly through the regulation of the αIIbβ3 and α2β1 integrins (77–80). A recent study described that platelet surface PDI enhances the ligand binding function of the platelet surface receptor GPIb alpha to promote platelet-leukocyte interactions (81). Apart from their postulated role in supporting platelet activation through these pathways, the specific substrates of extracellular thiol isomerases have mostly remained elusive.

**Figure 3:**
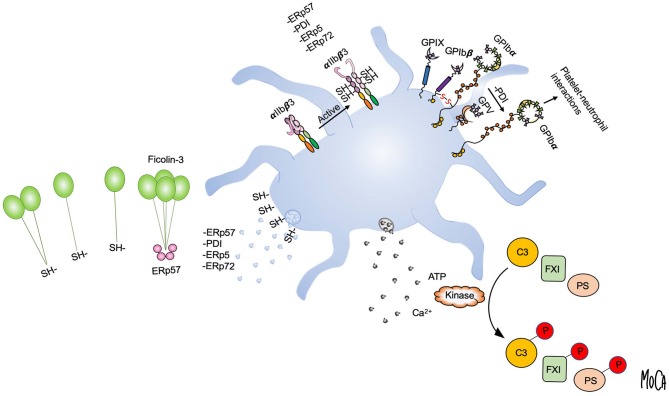
Post-translational protein modifications in the platelet microenvironment. Platelet activation and granule release create a microenvironment in which post-translational protein modifications can occur in the extracellular environment, as exemplified by disulfide bond rearrangement and phosphorylation. Platelet intrinsic reductase activity leads to exposure of free thiols and may provide a regenerating system for thiol isomerases. Platelets release vascular thiol isomerases that rearrange allosteric disulfide bonds in protein substrates. Multiple thiol isomerases are required for full α_IIb_β3 activation and platelet aggregation. Platelet-derived ERp57 cleaves multimeric ficolin-3 by disulfide bond reduction to limit platelet recognition by the lectin pathway (left). PDI released from platelets reduces disulfide bonds in GPIbα of the GPIb-IX-V complex to increase GPIbα-mediated platelet-neutrophil interactions (upper right). Exocytosis of platelet dense granules elevates the local ATP and Ca^2+^ concentrations. Substrates including complement C3, coagulation factor XI (FXI), and protein S (PS) can then be phosphorylated by platelet-derived or circulating kinases (lower right).

To gain insight into the mechanisms of action of extracellular thiol isomerases, we have developed a substrate-screening strategy based on an engineered variant of the enzyme that forms stable complexes with substrates (82). The application of this methodology to ERp57 led to the unanticipated finding that ERp57 displays a striking selectivity for the lectin pathway of complement among extracellular substrates (83). Specifically, ERp57 attenuates the activation of the ficolin-3-dependent branch of the lectin pathway and inhibits ficolin-3 ligand recognition, the proteolytic activity of the ficolin-3/MASP1 complex, and downstream complement activation. Like other lectin pathway PRMs, ficolin-3 forms large multimers of a basic subunit through intermolecular disulfide bonds, and a range of multimers and oligomers is found circulating in plasma (3). As compared to MBL and ficolin-2, which mostly assemble into 9-mers and 12-mers, we readily detected ficolin-3 18-mers and 21-mers in human plasma. By biochemical assays and mass spectrometry, we identified the mechanism by which ERp57 inhibits ficolin-3 and showed that ERp57 specifically cleaves large ficolin-3 multimers into smaller biologically inactive oligomers. This cleavage is the result of reduction of the intermolecular disulfide bonds that mediates ficolin-3 multimerization (Figure 3, left).

Our results also indicated that the unique susceptibility of ficolin-3 to disulfide bond cleavage is related to its extensive multimerization. Disulfide bonds under mechanical stress are primed for reduction, since application of force destabilizes the oxidized state of the disulfide (84, 85). In the case of ficolin-3, the assembly of large multimeric complexes would impose significant strain on the connecting disulfide bonds. We provided experimental support for this concept by determining their redox potentials, a measure of the stability of a disulfide bond. A clear multimerization-dependent effect was observed, in which the large biologically active ficolin-3 multimers appeared to be primed for reduction and displayed the highest redox potentials. Apart from explaining the preference of ERp57 for ficolin-3 among the lectin pathway PRMs, this multimer size-dependent redox potential may have general implications for the regulation of multimeric proteins.

The results detailed above were the first to describe a mechanism to regulate the lectin pathway at the PRM level, and how thiol isomerases can limit the recognition of platelets by PRMs. Thus, in addition to its role in thrombus formation, ERp57 appears to be a novel complement-regulatory protein, that could, for example, protect platelets from being targeted by the lectin pathway. Indeed, we have detected binding of ficolins and MASPs to activated platelets, with the ficolins tentatively recognizing epitopes that are exposed on the platelet membrane after activation (22). In combination with previous studies showing secretion of active C1-inhibitor from platelet alpha granules (33), our studies indicate that platelets appear to possess an intrinsic system to regulate their interactions with the lectin pathway.

## Platelet-Mediated Extracellular Phosphorylation

### Phosphate-Containing Plasma Proteins

Numerous plasma proteins, many of which are part of cascade systems, contain covalently bound phosphate *in vivo*. Using a colorimetric technique, we have found the amount of phosphate in plasma proteins to average 0.09 mol phosphate per mol of average protein in healthy blood donors (86, 87). Some of these plasma proteins were reported to be phosphoproteins decades ago, including fibrinogen (88), FV (89), complement component C3 (90–92), vitronectin (93), and human serum albumin (HSA) (94). Additional phosphoproteins have been identified only recently by mass spectrometric analysis of human plasma or serum samples (95–97). These include the complement components MASP-1, C5, C7, C8, and C9; the regulators factor I, factor H, C4BP, and clusterin; as well as the coagulation/fibrinolysis proteins FXIII, high molecular weight kininogen (HMWK), antithrombin (AT), and plasminogen (Table 2). Interestingly, several of these phosphoproteins were detected in serum but not in plasma, implying the involvement of platelets in the phosphorylation process, since serum is the end product of coagulation, a process that is initiated by platelet activation (see below).

**Table 2:** Complement- and coagulation-related plasma phosphoproteins.

	**Plasma phosphoproteins**					
	**Pre-proteomics**		**Plasma (96)**	**Serum (97)**	**Platelet-mediated phosphorylation**	
**COMPLEMENT**						
C3	x	(90–92)	x	x	x	(98–100)
C5				x		
C7				x		
C8			x			
C9			x	x		
Factor I			x	x		
Factor H			x	x		
C4BP				x		
MASP-1			x	x		
Vitronectin	x	(101)		x	x	(86)
Clusterin				x		
**COAGULATION/FIBRINOLYSIS**						
Fibrinogen	x	(88)	x	x	x	(86)
FV	x	(89)	x	x		
FXIII				x		
HMWK			x	x		
AT			x	x		
Plasminogen			x	x		
FXI					x	(102)
Protein S					x	(103)
**OTHERS**						
HAS	x	(94)	x	x		(86)
α2-MG			x			

*C4BP, C4b-binding protein; MASP-1, MBL-associated serine protease 1; F (coagulation) factor; HMWK, high molecular weight kininogen; HSA, human serum albumin; α2-MG, α2 Macroglobulin*.

*The table gives an overview of phosphorylated plasma proteins involved in complement activation and coagulation that have been reported in the literature. Pre-proteomic studies indicate targeted investigations performed before mass spectrometry became available. The table also shows phosphoproteins detected in unbiased mass spectrometry analyses of plasma and serum samples*.

### Plasma ATP

The phenomenon of extracellular phosphorylation has been known for decades, and numerous extracellular kinases (ectokinases) have been identified; however, the physiological relevance of extracellular phosphorylation, at least in the plasma milieu, has been questioned. Nevertheless, the concept of extracellular phosphorylation has recently regained interest in the era of proteomics, since it has been demonstrated by high-throughput mass spectrometry and other techniques that numerous extracellular proteins, both in tissue and in body fluids, can exist in phosphorylated forms (104, 105).

A key point is whether, under normal conditions, plasma contains sufficient amounts of ATP. EDTA-plasma drawn from healthy volunteers who had received no medication for at least 10 days has been reported to contain between 0.2 and 1 μM ATP (98, 106). (EDTA inhibits ATP catabolism and is routinely used for ATP determinations). In blood collected in EDTA with other additives such as theophylline and dipyridamole to inhibit ATP release from the blood cells, the detected levels are even lower, typically 28–650 nM (98, 106–108). Since the Km for ATP for classical protein kinases such as cyclic AMP-dependent protein kinase (PKA) are in the range of 13–30 μM (109, 110), it is unlikely that any substantial amount of extracellular phosphorylation takes place in plasma in the absence of cellular activation and/or release. However, ATP and Ca^2+^ in high amounts [1–2 M (111)] are found in the dense granules of platelets. When these granules are released in response to physiological activation by agonists such as thrombin or ADP, the plasma concentration of ATP increases from 1 to 20 μM as measured in activated PRP (98), and similar levels are found in the blood after vascular damage (112). This concentration, which should be enough to support phosphorylation, would be expected to be even higher in close proximity to activated platelets. In addition, cells such as lysed erythrocytes (106) or dying or apoptotic cells (113, 114) may also show an increased plasma concentration of ATP; however, in contrast to platelet-mediated release, this increase is the result of a non-regulated process.

Regardless of its cellular origin, ATP and Ca^2+^ can be utilized by various protein kinases (either released from the platelets or already present in the plasma, originating from other cells or exposed on their surfaces) to phosphorylate plasma proteins in the micro-milieu adjacent to the platelets. In this context, it should be taken into account that a number of plasma proteins are phosphorylated by several protein kinases, with different impacts on protein function (see below).

### Extracellular Protein Kinases

The nucleus of this review article concerns the interaction between activated platelets and the complement system. Therefore, in this section we will focus on protein kinases identified as being present in or released from platelets. The postulated origin of specific protein kinases found in the plasma that can mediate extracellular phosphorylation is discussed in detail in Yalak and Vogel (104) and Klement and Medzihradszky (105).

Protein kinase activities in the form of protein kinase A (PKA) (93), several isoforms of protein kinase C (PKC) (115), and casein kinases 1 and 2 (CK1 and CK2) (98–100, 102, 103, 107–115) have all been identified in the releasate from activated platelets. They have all been shown to mediate extracellular phosphorylation of specific plasma proteins, in particular proteins within the cascade systems, provided that ATP and Ca^2+^ are present in sufficient amounts. These protein kinases are all classified as serine/threonine kinases (116). Recently, a newly identified kinase, designated vertebrate lonesome kinase (VLK), was the first protein-tyrosine kinase shown to be released from TRAP-activated platelets (117, 118). Through proteomic approaches, protein kinases of all these classes have been identified in platelets (119). Information from platelet releasate is less conclusive regarding the identification of protein kinases (71, 120), suggesting that they may be associated with platelet-derived microparticles rather than being found in the fluid phase.

### Examples of Plasma Proteins Whose Function Is Affected by Phosphorylation

Most of the initially identified human plasma phosphoproteins (fibrinogen, C3, vitronectin, FV) have been shown to be subject to phosphorylation *in vitro* by various protein kinases with different functional effects.

Fibrinogen is a substrate for PKA, PKC, CK1, and CK2 (121), and phosphorylation affects its activation by thrombin, leading to changes in the thickness of the resulting fibrin bundles. Similar alterations are found in plasma collected from patients after hip replacement operations, i.e., in a situation in which platelets have been activated *in vivo* (122).

Complement component C3 is a substrate for at least five different protein kinases (including platelet CK, which is discussed below). All of these covalently bound phosphate groups affect C3's activity in distinct ways. PKA, PKC, and an ecto-kinase from the parasite *Leishmanina major* all phosphorylate a serine residue in the C3a moiety, thereby protecting C3 from activation (123, 124). In contrast, C3 phosphorylated by CK2 derived from the leukemia cell line U937 (either intracellularly prior to release or extracellularly) is markedly more sensitive to proteolytic cleavage, generating iC3b (92).

Similarly, vitronectin is phosphorylated by PKA (93), PKC, and CK2. Phosphorylation by PKC attenuates its cleavage by plasmin (125), whereas phosphorylation with CK2 enhances cellular spreading (126).

Plasma C9 becomes phosphorylated by CK2 ectokinases on human leukemia cell lines such as U937 or K562, with the net effect of inhibiting complement-mediated cell lysis (127).

The phosphate content of fibrinogen, vitronectin, C3, and HSA has been shown to increase in the plasma after platelet activation (86), further underscoring the importance of platelets as mediators of extracellular phosphorylation but without addressing the question of whether their main effect is to provide protein kinase(s) or enough ATP to sustain phosphorylation, or a combination of both.

It is possible that HSA, the most abundant plasma protein and a substrate for multiple protein kinases (94), can act as a scavenger phosphorylation substrate in plasma.

### Plasma Proteins Whose Activity Is Regulated by Platelet-Mediated Phosphorylation

A direct link between platelet activation, extracellular phosphorylation of plasma proteins, and alteration of the proteins function(s) has been demonstrated only in a few cases, such as complement component C3, coagulation factor XI (FXI), FVa, and, most recently, the anticoagulant effector protein S (Figure 3, right).

In a series of publications, we have reported that extracellular phosphorylation of C3 is mediated by a putative casein kinase associated with activated platelets. The main phosphorylation site was a serine residue in the C3d, g portion of the α-chain, and phosphorylation had dramatic effects on at least three of C3's functions, with the combined effect being an enhancement of the complement-mediated opsonization of immune complexes resulting from C3 phosphorylation. First, we found that the phosphorylation of C3b increases its resistance to cleavage by factor I to iC3b (98). Because C3b (but not iC3b) is a subunit of the alternative pathway C3 convertase, the activation of C3 is increased, as we were able to demonstrate in the form of an enhanced opsonization of model immune complexes (99). Lastly, we showed that bound phosphorylated C3b has a significantly higher affinity for CR1 than does its unphosphorylated counterpart (100), suggesting that phagocytosis of the opsonized immune complexes may be facilitated by platelet-mediated phosphorylation.

We further showed that the same platelet-derived casein kinase phosphorylates FXI in the coagulation cascade. Phosphorylation markedly enhanced FXI activation by FXIIa in particular, but also to a lesser extent by thrombin, suggesting that the phosphorylation enhances the activation of the coagulation cascade induced by the contact system and facilitates the thrombin-meditated amplification loop of the coagulation system, which involves the intrinsic components starting with FXI (102). The concept of increased activation was further supported by our observation that the levels of FXIa-AT complexes (which are generated secondarily to FXI activation) were higher in plasma from systemic lupus erythematosus (SLE) patients than in controls and that there was a clear correlation between the levels of FXIa-AT complexes and β-thromboglobulin, a marker for platelet activation (87).

Furthermore, it has been suggested that anticoagulation is regulated by platelet-mediated phosphorylation: Coagulation factor Va becomes more readily inactivated by activated protein C after phosphorylation by platelet casein kinase (128). More recently, platelet-secreted casein kinase(s) have been shown to phosphorylate protein S, thereby enhancing its activated protein C cofactor activity (103). In that publication, the authors postulated that extracellular platelet-mediated phosphorylation of protein S is a previously unrecognized mechanism for regulating its anticoagulant activity, and that role of phosphorylation most likely applies to all the substrates discussed here.

## The Pro-Coagulant Platelet Phenotype in Disorders of Complement Regulation

Given the numerous and complex interactions between complement and platelets, it is interesting to note that conditions with a genetically determined deficiency in complement regulation include a pro-thrombotic platelet phenotype in their clinical presentation. Here we further discuss two such conditions, atypical hemolytic uremic syndrome (aHUS) and paroxysmal nocturnal hemoglobinuria (PNH), and consider what they can teach us about platelet-complement interactions.

### Atypical Hemolytic Uremic Syndrome (aHUS)

aHUS is a thrombotic microangiopathy that presents with a symptom triad of intravascular hemolysis, thrombocytopenia, and acute kidney failure caused by complement-mediated tissue damage (129). aHUS appears to primarily be caused by a genetically determined impaired regulation of the alternative pathway, and heterozygosity for mutations in the alternative pathway regulator factor H is the most prevalent genetic lesion in aHUS. Mutations in factor I, CD46, factor B, and C3 have also been consistently observed, all of which have a common consequence, alternative pathway dysregulation (130, 131). The penetrance of aHUS causing mutations is not complete and often an external trigger such as an infection is needed to precipitate the disease in individuals with genetic predisposition (129).

Platelet consumption is an important but underappreciated feature of aHUS, where complement attack is postulated to cause platelet activation and formation of platelet-rich microthrombi and eventually thrombocytopenia. aHUS-associated factor H mutations cluster in the C-terminal part of the protein and impair binding to self surfaces, with insufficient protection from autologous complement attack as a result (132, 133). It has been shown that aHUS-associated factor H mutations directly impair platelet binding, resulting in increased complement deposition on platelet surfaces, increased baseline platelet activation, and formation of pro-coagulant microparticles (134). Further evidence for a prothrombotic state associated with platelet activation has been obtained in a mouse model of aHUS, in which a factor H point mutation found in aHUS patients has been introduced into mice (135). In addition to renal microangiopathy and kidney failure, mice harboring the mutant factor H variant developed an unexpectedly severe phenotype, with systemic thrombophilia, large vessel thrombosis, and multi-organ involvement.

### Paroxysmal Nocturnal Hemoglobinuria (PNH)

PNH is a hemolytic anemia caused by complement-mediated hemolysis of red blood cells (136). It has a different genetic background than does aHUS and is caused by somatic mutations in clones of hematopoietic cells, leading to a deficiency of glycosylphosphatidylinotisol (GPI)-anchored proteins, and thus a lack of the GPI-linked complement regulators CD55 and CD59. The primary targets of complement attack in PNH are GPI-deficient red blood cells, which are continuously consumed by intravascular hemolysis. PNH is also associated with an increased risk of thrombosis, to which excessive complement-mediated platelet activation has been hypothesized to contribute. Platelets from GPI-deficient PNH clones still have some complement-regulatory capacity, since they express CD46 and recruit circulating factor H to the cell surface, as discussed above. Nonetheless, it appears that these soluble complement regulators are not sufficient to prevent complement attack in the context of the loss of CD55 and CD59, and there is clear evidence of complement-mediated platelet damage in PNH. Platelet counts are low, and both platelet dysfunction resulting from chronic hyperstimulation (137) and higher levels of baseline platelet activation have been observed (138). Interestingly, elevated numbers of platelet-derived microparticles are found in PNH patients, an observation that provides intriguing evidence of complement-mediated platelet microparticle formation (139).

### Mechanisms of the Prothrombotic State in Disorders of Complement Regulation

Introduction of the anti-C5 antibody eculizumab (Soliris®), which blocks MAC formation and C5a generation, has constituted a therapeutic revolution for patients with aHUS and PNH. Anti-C5 treatment provides a clear benefit in these patients, and today, although very expensive, it is still the treatment of choice (140). Interestingly, eculizumab treatment rapidly normalizes suppressed platelet counts in both aHUS (141) and PNH (142) patients, indicating that platelet consumption in these conditions is complement-mediated. Because eculizumab also significantly reduces thromboembolic events, there appears to be a causal involvement of the terminal complement pathway in the hypercoagulable state observed in these patients (143).

What is the mechanism by which complement activates platelets and converts a quiescent vasculature to a prothrombotic state in these conditions? Direct complement-mediated damage to endothelial cells and platelets likely plays a major role, as well as sublytic concentrations of C5b-9 that potently triggers cellular activation. Apart from effects mediated by direct complement deposition, release of anaphylatoxins is also a potential contributor. C3a exerts stimulatory effects on platelets through the C3a receptor (44, 45). PAR1 and PAR4 have recently been identified as receptors for C4a (144), and although platelets were not directly tested in the same study, these PARs are strong agonistic platelet receptors that could implicate C4a as a mediator of platelet-complement cross-talk. The C5a-C5aR1 signaling axis mediates a potent pro-inflammatory response, but with the main target cells being endothelial cells and leukocytes. Blood clotting induced by intravascular invasion of pathogens has been shown to be dependent on C5 and the induction of tissue factor expression on monocytes. A strongly diminished response to LPS or whole bacteria was seen in blood from an individual with congenital C5 deficiency, a defect that was phenocopied in normal blood by addition of eculizumab or a C5aR1 antagonist, implicating signaling by soluble C5a rather than MAC deposition in this scenario (145, 146). Likewise, in a follow up study on an aHUS mouse model based on factor H deficiency, breeding of the mice onto a C5aR1 deficient strain rescued the large vessel thrombosis phenotype, while thrombotic microangiopathy and renal damage was MAC dependent. Interestingly, thrombocytopenia was seen in both scenarios, indicating that both C5a-dependent leukocyte activation and increased MAC formation contributes to a procoagulant platelet phenotype. Ongoing clinical trials of C5a-directed therapeutics and C5a receptor antagonists will undoubtedly provide important insights into these phenomena (140).

Collectively, aHUS and PNH demonstrate that in the context of a deficiency in its regulation, complement is clearly a very potent trigger of thrombotic events. The question remains as to what extent complement activation is a physiological mechanism that supports platelet activation and thrombosis, and whether enough complement activity can be generated on cells to trigger these events under conditions of full complement-regulatory capacity.

## Conclusions

There is now ample evidence to consider the platelet an immune cell and not limit its role to primary hemostasis and platelet plug formation. Platelets interact with the cascade systems of blood in a highly controlled fashion, with complement mediating platelet-leukocyte interactions, contributing to platelet activation, and helping to mount a pro-inflammatory response, all while preserving host cell integrity and avoiding complement-mediated damage to healthy cells.

The platelet phenotype of disorders of complement regulation involving excessive platelet activation and thrombocytopenia resulting from platelet consumption provide intriguing evidence regarding how complement can trigger platelet activation. However, the question remains: Do numerous complement regulators serve to totally prevent complement activation in the platelet microenvironment under physiological conditions? Investigators need to determine how this mechanism operates under physiological levels of complement-regulatory capacity and reconcile this model with the abundance of complement regulators associated with platelets.

Post-translational modifications of proteins, thought to occur exclusively in the intracellular milieu, are now gaining in interest as important regulatory mechanisms throughout the entire lifespan of a secreted protein. Mass spectrometry and proteomic approaches have been instrumental in characterizing and quantifying these mechanisms for fine-tuning protein function. In the context of platelets, we are beginning to unravel how these chemical reactions direct platelet activation and the interaction between platelets and the complement and coagulation systems. The concept of a “platelet microenvironment,” in which exocytosis of platelet granules provides the co-factors and catalytic enzymes that drive these reactions, provides a model for how disulfide bond modifications and extracellular phosphorylation reactions proceed in the extracellular space, though many of the specific enzymes and substrates involved remain to be identified.

## Author Contributions

All authors listed have made a substantial, direct and intellectual contribution to the work, and approved it for publication.

### Conflict of Interest Statement

The authors declare that the research was conducted in the absence of any commercial or financial relationships that could be construed as a potential conflict of interest.
